# Biomechanical Analysis of Rheumatoid Arthritis of the Hand and the Design of Orthotics: A Finite Element Study

**DOI:** 10.3390/bioengineering12050462

**Published:** 2025-04-27

**Authors:** Guiyuan Li, Jie Yang, Pengfei Feng, Xiaona Li, Weiyi Chen

**Affiliations:** Institute of Biomedical Engineering, College of Artificial Intelligence, Taiyuan University of Technology, Taiyuan 030024, China; liguiyuan8725@163.com (G.L.); yyyangdwy@163.com (J.Y.); lixiaona@tyut.edu.cn (X.L.); chenweiyi@tyut.edu.cn (W.C.)

**Keywords:** rheumatoid arthritis (RA), hand orthosis, finite element analysis, biomechanics, three-point force

## Abstract

Hand orthoses are often recommended as a rehabilitation measure for patients with rheumatoid arthritis (RA). However, existing research on the efficacy of hand orthoses has predominantly focused on 3D-printed devices and post-intervention clinical functional assessments, which tend to be subjective. There is a notable lack of biomechanical studies evaluating the effects of wearing orthoses. Therefore, in this study, the finite element method was used to analyze the biomechanical properties of an RA hand. A hand orthosis was designed based on the principle of three-point force, and a composite model of the RA hand and orthosis was constructed to verify its effectiveness. The results showed that the peak stress and displacement of the RA hand were 3.22–183.21% and 28.81–124.23% higher than those of the normal hand. Compared with the RA hand under direct force, the peak stress of the RA hand after wearing orthosis was generally reduced by 3.05–55.60%, and the peak displacement was generally reduced by 20.35–71.43%, verifying the effectiveness of the orthosis. Additionally, variations in the magnitude of three-point forces influenced the orthopedic effects. This study proves the effectiveness of hand orthosis and provides some theoretical data for subsequent research and treatment of rheumatoid arthritis.

## 1. Introduction

Rheumatoid arthritis (RA) is a prevalent chronic autoimmune disease characterized by progressive joint damage and inflammation [1], which involves insidious cartilage and bone destruction, leading to a high disability rate. The main clinical manifestations are persistent pain and swelling of the affected joints [2,3]. Notably, approximately 75% of RA cases affect the wrist and fingers [4]. A literature review has indicated that joint diseases are the leading cause of physical disability, with RA being the most significant contributor to disability rates [5]. Rheumatoid ulnar deviation of the metacarpophalangeal joint is one of the most common deformities associated with hand RA. This condition affects the distal ends of the four fingers, excluding the thumb, with the metacarpophalangeal joint acting as the axis of lateral deviation. Unlike the reduced mobility caused by lower limb dysfunction, upper limb function cannot be replaced by assistive devices such as crutches and wheelchairs [6]. If not prevented in time, it will directly affect the patient’s self-care ability and reduce the quality of life of the patient [7].

At present, there is no complete cure for RA, which is a clinically refractory disease [8]. Therefore, early diagnosis and treatment are of great significance for RA patients [9,10]. Current treatment options for rheumatoid arthritis include pharmacotherapy, surgical intervention, and rehabilitation therapy [11]. Among these, pharmacotherapy offers rapid onset of action, but is frequently associated with significant adverse effects. Surgical intervention is generally recommended for patients in the moderate to advanced stages of the disease; however, it is highly invasive and carries substantial risks, such as bleeding, infection, and other complications [12,13]. Rehabilitation treatment can develop different stable rehabilitation programs according to the different periods of the patient’s disease. Specifically, hand orthosis rehabilitation therapy can enhance the overall therapeutic effects of RA to a certain extent. The use of orthotics to alleviate pain and improve hand function is a common clinical practice for RA patients. This approach allows patients to effectively reduce pain and enhance their quality of life [14].

Current research on hand orthoses predominantly focuses on structural design optimization, 3D printing technologies, and post-intervention clinical follow-ups. However, the acquired data remain largely subjective, lacking biomechanical analyses of orthosis-hand interactions during wear. Moreover, the three-dimensional design of hand orthoses often relies on empirical knowledge from researchers or orthotists, resulting in inconsistent clinical correction outcomes and ongoing debates regarding efficacy. Furthermore, while the three-point force correction principle has been extensively applied to foot and spinal orthotics, its integration with hand orthoses for RA management remains underexplored. To address these gaps, this study established three-dimensional finite element models of both healthy and RA-affected hands through literature-informed methodologies. Utilizing the healthy hand model as a control, we investigated the biomechanical behavior of RA hands via finite element analysis. Tailored to RA-specific pathologies—including ulnar deviation, joint swelling, and hyperflexion of interphalangeal joints—a personalized orthosis was designed by applying multiple three-point force configurations aligned with rehabilitation principles. The orthosis was then virtually integrated with the RA hand model to simulate wear, enabling biomechanical evaluation of its corrective effects through computational simulations.

## 2. Materials and Methods

### 2.1. Research Preparation

Two volunteers were recruited for the purpose of obtaining computed tomography images of the hand, wrist, and related musculoskeletal structures. One was a healthy person, and the other was a patient with early rheumatoid arthritis. The scanning range encompasses the entire length of the bone, from the mid-radius and ulna to the distal phalanx, conducted continuously. The image resolution was 512 × 512, and the data were saved in DICOM 3.0 format. The implementation of the research scheme in this paper met the relevant ethical requirements of Taiyuan University of Technology and was reviewed by the Biological and Medical Ethics Committee (No. TYUT2024122302).

Software: Mimics 19.0 (Leuven, Belgium), Geomagic Studio 2013 (Research Triangle Park, NC, USA), SolidWorks 2021 (Waltham, MA, USA), and ANSYS Workbench 2021 R1 (Canonsburg, PA, USA).

### 2.2. Three-Dimensional Model Reconstruction of the Hand

Computed tomography (CT) images of healthy and rheumatoid arthritis hands were imported into Mimics software for detailed processing. Key operations, including threshold segmentation, region growing, and removal of disconnected components, were applied to generate preliminary models of the hand bones and soft tissues. To ensure the accuracy of the surface generation and subsequent finite element analysis, the 29 hand bones—comprising the radius, ulna, carpals, metacarpals, and phalanges—were carefully isolated. Three-Dimensional models were reconstructed using the “Calculate 3D” and “Smooth Mask” commands. The resultant bone and soft tissue models for both the healthy and RA groups were then imported into Geomagic Studio software. Advanced techniques such as remeshing, smoothing, and precise surface modeling were employed to create high-quality surface solid models. Finally, the solid models were imported into SolidWorks software, where operations including origin alignment, sketching, creation of offset surfaces, component assembly, and cartilage modeling were performed to obtain a complete assembly of both the healthy and RA hand models.

### 2.3. Personalized Design of Hand Orthosis

**Figure 1:**
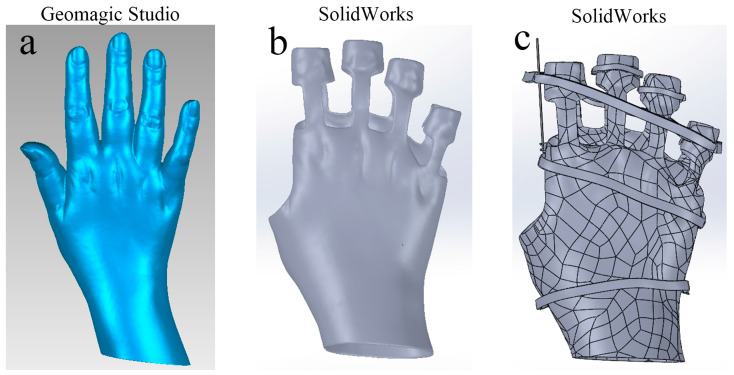
Hand orthosis modeling process. Figure (**a**) shows the 3D model of the external contour of the hand in rheumatoid arthritis (RA), (**b**) shows the simplified orthosis model used for subsequent finite element analysis, and (**c**) shows the final orthosis model.

The 3D model of the hand profile from the patient with rheumatoid arthritis was imported into Geomagic Studio software for further processing, as shown in Figure 1a. Initially, the model of the orthosis was segmented along the coronal plane into two main sections: the upper and lower parts. In consideration of the anatomical features and functional limitations associated with RA, the surface was further divided at specific anatomical landmarks, including the distal ends of the five fingers, the distal portions of the radius and ulna, the metacarpophalangeal joints, and the region between the proximal interphalangeal joints. This process yielded a preliminary orthosis model.

Subsequently, the entire structure was offset outward by 0.2 mm. Although an outward offset of 2–3 mm would typically be more suitable to accommodate padded gauze, a 0.2 mm shift was selected to facilitate the subsequent finite element analysis while ensuring appropriate contact with the soft tissues. Although increasing the shell thickness would generally be advisable, special attention was given to the proximity of the third and fourth fingers in this model. If the shell were excessively thick, it could cause interference between the near-knuckle phalanges of these fingers. To mitigate this risk, the shell thickness was reduced by 1 mm at the region between the third and fourth fingers. The polygonal model was then converted into a three-dimensional digital representation using advanced surfacing techniques to ensure accuracy and precision in the final model.

The aforementioned 3D digital models were imported into SolidWorks software. Since one of the symptoms of rheumatoid arthritis is ulnar deviation, a groove and a support bar close to the hand were first drawn near the second metacarpophalangeal joint. Subsequently, Velcro straps were applied to the second to fifth proximal interphalangeal joints, pulling them towards the radial side. Three Velcro strips were then affixed at the proximal interphalangeal, metacarpophalangeal, and radial carpal joints. These strips allowed for the correction of deformities by adjusting their tightness according to the patient’s condition, following the principle of three-point force correction. Furthermore, considering rheumatoid arthritis’s symptom of joint swelling and abnormal flexion or extension of the proximal interphalangeal joints, the dorsal section of the orthosis’s second to fifth proximal interphalangeal joints was thickened. Concurrently, Velcro was affixed to the proximal interphalangeal joints of the second to fifth fingers. The combination of the thickened portion and the Velcro allows for the formation of multiple sets of three-point forces at the proximal interphalangeal joints, which can be used to correct the patient’s hand. The final design of the hand orthosis is shown in Figure 1c, while the modeling process is illustrated in Figure 1.

The curved design of the orthosis ensures a precise fit to the human hand, thereby enhancing its fixation effect. Additionally, the configuration of the notch, support bar, and Velcro straps can be adjusted in real-time, making it a suitable option for dynamic orthopedic procedures. By concurrently applying multiple three-point forces, discomfort can be alleviated more effectively and deformities can be corrected. In subsequent finite element analyses, the orthopedic effect of the taping force will be represented through the application of a three-point force load in Workbench software. To avoid stress concentration and simplify the model, a composite model was con-structed by integrating the orthotic model (excluding the support bar and Velcro straps as shown in Figure 1b) with the RA hand model for subsequent finite element analysis.

### 2.4. Finite Element Modeling

SolidWorks software was used to reconstruct the normal bone, normal soft tissue, RA bone, RA soft tissue, and hand orthosis, which were then assembled to generate three models: the normal hand model, the RA hand model, and a composite model combining the RA hand model with the orthosis. These models were subsequently imported into ANSYS Workbench for static analysis.

For material property assignment, the cortical bone, cancellous bone, soft tissue, and articular cartilage of the hand were treated as isotropic linear elastic materials, while the hand orthosis was modeled as polylactic acid [15]. As reported in clinical studies, the three primary factors contributing significantly to the pathological processes of the wrist in RA are synovial hyperplasia, cartilage destruction, and ligamentous laxity. Based on these established criteria, the RA model in this study was constructed [16]. To simulate the reduced bone density associated with synovial hyperplasia, the elastic modulus of the bone was reduced accordingly. Specifically, the bone density was reduced by 33% for normal cortical bone and 66% for cancellous bone in the context of RA. To represent the most severe case of cartilage destruction, the entire articular cartilage was removed from the bone. Finally, to model ligament laxity, the ligaments were represented by spring unit simulations, with normal ligament stiffness values derived from the work of MN Bajuri et al. [17,18,19,20]. For the RA model, the ligament stiffness was set to 50% of the normal ligament stiffness. The material properties for the hand finite element model and the orthotic material were assigned based on values obtained from the literature, as summarized in Table 1.

Contact settings were defined in the Workbench software, where potential contact pairs were automatically generated by adjusting the tolerance value. Among these, joint contacts were specified as frictional contacts. The friction coefficient for healthy joints was set to 0.02, while for the RA joints, it was set to 0.3 [17]. To simplify the model, all other contacts were classified as bonded contacts. The selection of target and contact surfaces was determined based on mesh stiffness and density, with the coarser or stiffer surface designated as the target, and the finer or more flexible surface as the contact. Based on the referenced study, the relationships among the orthosis-soft tissue, soft tissue-cortical bone, and cortical bone-cortical bone interfaces were established, with the orthosis and cortical bone as target surfaces, and the soft tissue and cancellous bone as contact surfaces.

Grid Division. The type and size of meshing in the Workbench software directly influence both the duration of calculation time and the accuracy of the results. Therefore, it is very important to select the appropriate meshing precision for finite element analysis. To enhance mesh quality while accounting for the irregularity and complexity of hand bones and soft tissues, tetrahedral meshing was employed. Based on the research objectives and prior experience, an initial mesh division was performed, followed by evaluation and diagnosis of mesh quality. The mesh size was iteratively adjusted until optimal values were achieved. Ultimately, the mesh sizes were set as follows: 1 mm for bone, 0.5 mm for cartilage, 3 mm for soft tissue, and 3 mm for orthotics. The Element Quality index, which evaluates the relationship between volume and side length (with 0 representing the worst quality and 1 representing perfect quality), yielded a maximum value of 1 and an average value of 0.81756. These results indicated that the meshing outcome was ideal and the cell shapes were reasonable. Additionally, the Aspect Ratio, defined as the ratio of the longest side to the shortest side of the unit, ideally should be less than 5, with a recommended upper limit of 10. The results demonstrated a minimum value of 1.1648 and an average value of 1.9386, confirming that the mesh quality fell within the ideal range. Overall, the mesh evaluation results confirmed that the chosen mesh size was appropriate and the mesh quality was high, thereby enhancing the accuracy and convergence of subsequent finite element analysis results. After the calculation, the total number of nodes and elements were as follows: the normal model contained 1,990,878 nodes and 1,262,481 elements; the RA model included 1,440,738 nodes and 889,147 elements; and the composite RA hand-orthotic structure consisted of 1,501,018 nodes and 917,995 elements.

As for boundary condition settings, to facilitate convergence of the solution, the proximal ends of the radius and ulna were fully restrained. The stops of the carpometacarpal joints and some of the tendons (Abductor pollicis longus, flexor carpi radialis, flexor carpi ulnaris, extensor carpi ulnaris, extensor carpi radialis brevis, and extensor carpi radialis longus) were fixed to prevent movement in the x- and y-directions, thereby ensuring that the bones would move in the direction of the applied load [17].

Regarding load setting, four motions—namely, hand flexion, extension, radial deviation, and ulnar deviation—were simulated [21,22], with the applied loads for both the normal and RA models outlined in Table 2. The load values measured in non-arthritic hand volunteers were set to 100%. However, patients with rheumatoid arthritis (RA) may not be willing or capable of enduring higher stress levels. Therefore, stress levels of 50% and 10% were chosen for this study. Corresponding loads were applied to both the normal and RA models. Constraints were applied to the distal radius and ulna to fixate them, and the simulation was then solved to generate stress and displacement contour maps. These maps encompassed stress, total displacement, and directional displacement, allowing for the observation of biomechanical differences between the normal and RA models.

**Figure 2:**
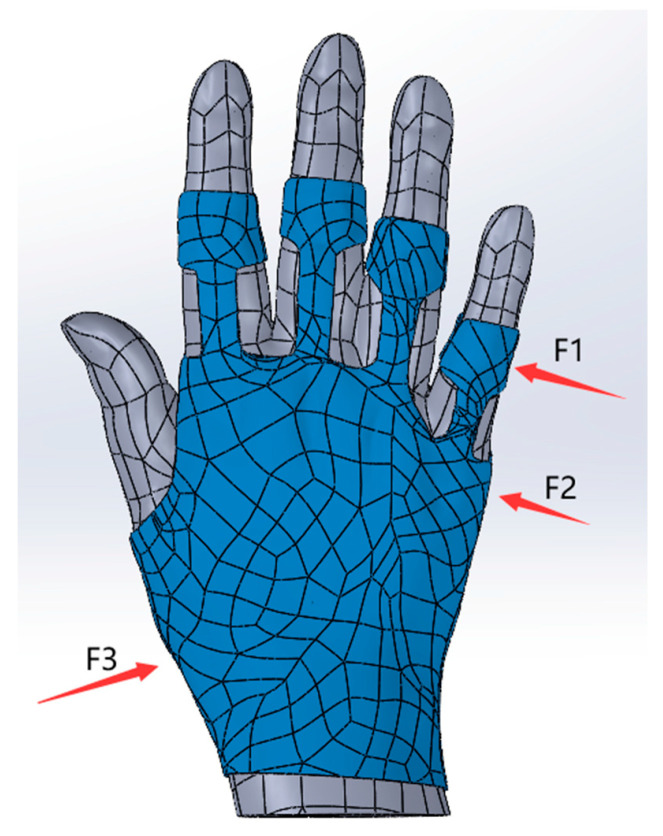
Three-point force correction force diagram of the rheumatoid arthritis (RA) hand. The three points of force application are as follows: F1 is the ulnar side of the fifth proximal interphalangeal joint; F2 is the ulnar side of the metacarpophalangeal joint; and F3 is the radial side of the radial wrist joint.

Finite Element Analysis of Three-Point Force Correction: A three-point force analysis model for RA was employed to evaluate the virtual stress and displacement changes before and after the application of the hand orthosis. In consideration of the patient’s condition, slight ulnar deviation and hyperflexion of the fifth finger were observed. To simplify the analysis, this study focuses on three-point force correction at 50% flexion and 50% radial deviation. The boundary conditions and the flexion and radial deviation movements were identical to those described previously. Subsequently, the RA composite model was subjected to a three-point force in the coronal plane, in line with the aforementioned working conditions. This approach aimed to determine whether the application of a three-point force could effectively correct the deformities in RA patients when using the hand orthosis. The three-point forces in the coronal plane were applied at the ulnar side of the fifth proximal interphalangeal joint, the ulnar side of the metacarpophalangeal joint, and the radial side of the radial carpal joint, as illustrated in Figure 2 (F1, F2, and F3). According to the literature [23], human skin’s pressure tolerance is 2.5 N/cm^2^. Additionally, clinical experience suggests that the general corrective force does not exceed 10 N. Therefore, in the subsequent finite element analysis, the magnitude of the three-point force was adjusted to evaluate the orthopedic effect of the hand orthosis on RA patients, while adhering to the aforementioned limits.

## 3. Results

### 3.1. Normal and Patient Hand Models

**Figure 3:**
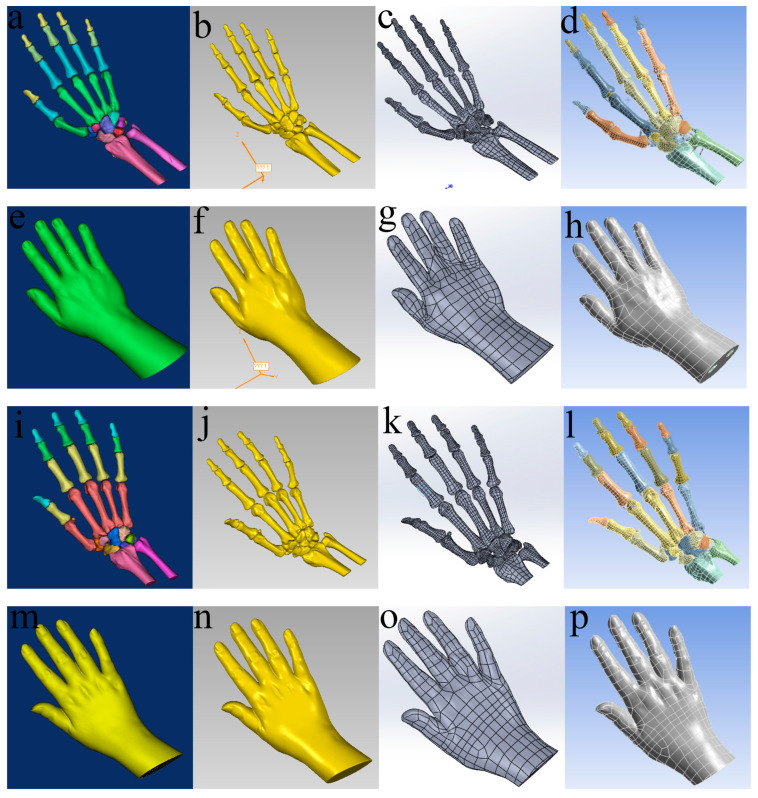
Models of the normal hand and hand of an RA patient. (**a**–**d**), (**e**–**h**), (**i**–**l**), and (**m**–**p**) show the models obtained via Mimics, Geomagic Studio, SolidWorks, and Workbench software for normal hand bone, normal hand soft tissue, RA hand bone, and RA hand soft tissue, respectively.

CT images of both healthy subjects and individuals with rheumatoid arthritis were imported into the Mimics software to generate geometric models of the normal hand and the RA hand, respectively. These models were then imported into Geomagic Studio for optimization and the generation of NURBS (Non-Uniform Rational B-Spline) surface sheets. Subsequently, the models were transferred to SolidWorks for the assembly of components and the creation of articular cartilage. The finalized geometric models were imported into ANSYS software for preprocessing, including the establishment of ligaments and meshing. Loads and boundary conditions were then applied, and finite element analysis was performed. The models of the normal and RA hands are presented in Figure 3.

### 3.2. Validation of Model Validity

**Figure 4:**
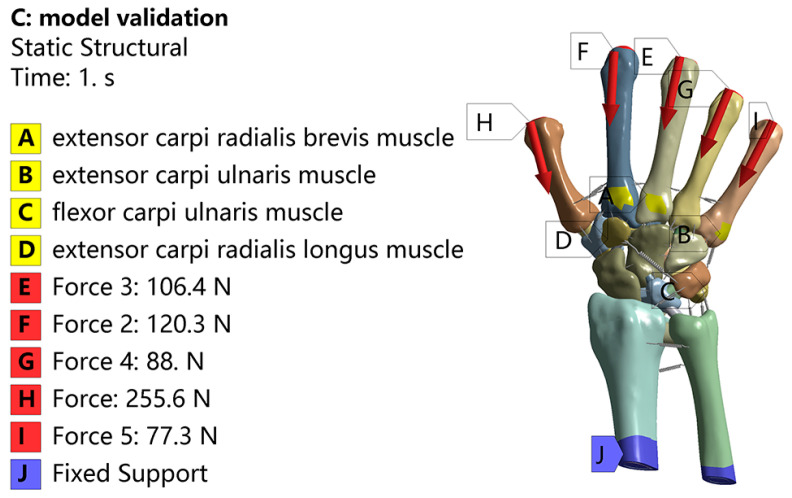
Model validation force diagram.

Before conducting the finite element analysis, model validation was performed in this study. In accordance with the studies by MN Bajuri and Magnús K. Gíslason et al. [17,24], loads of 255.6 N, 120.3 N, 106.4 N, 88.0 N, and 77.3 N were applied to the distal ends of the first through fifth metacarpal bones, respectively (see Figure 4). Upon completion of the calculations, the load transfer ratio on the radius and ulna for the normal finite element model was determined to be 81.5% and 18.5%, respectively. For the normal model, the load transfer ratio on the radius and ulna ranged from 78.7% to 92.8% and from 7.2% to 21.3%, respectively, which are within the values reported in the literature. In the RA model, the load transfer ratio on the radius and ulna was 76.1% and 23%, respectively. The increased load transfer to the ulna can be attributed to the ulnar deviation in the carpal tunnel of RA patients, which causes the dislocation of carpal bones towards the ulnar side, resulting in elevated ulna load transfer. This finding was consistent with the simulation results, which indicated a higher proportion of load transfer to the ulna. The statistical data results are presented in Table 3. Therefore, the effectiveness of the model can be verified by comparing with the published literature data.

### 3.3. Biomechanical Analysis of Hand Models

**Figure 5:**
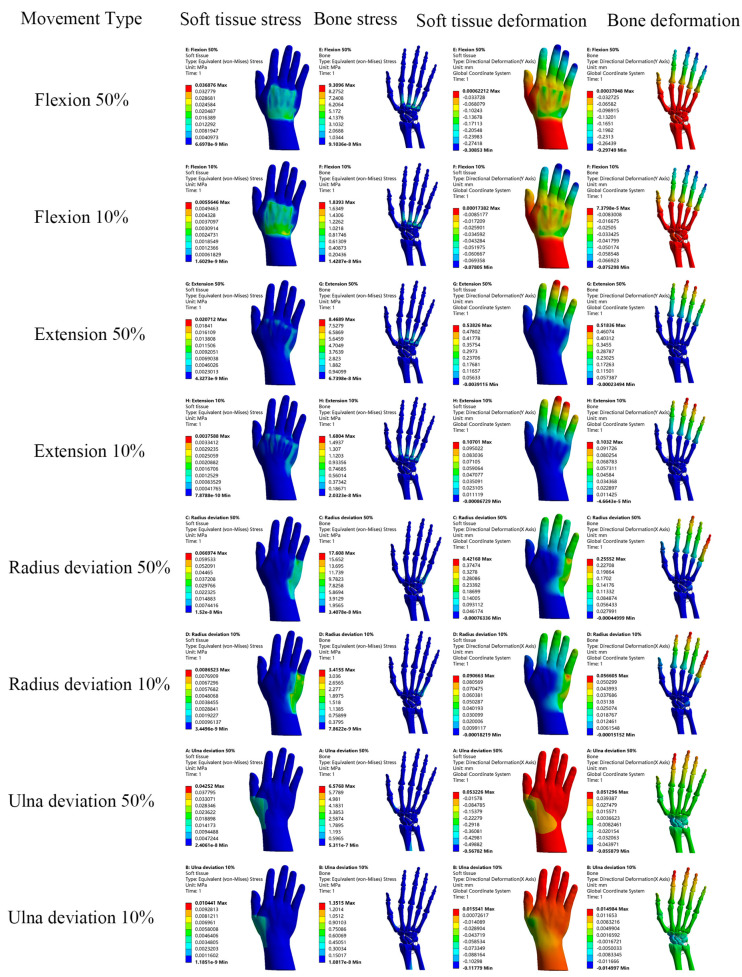
Stress and displacement cloud of normal hand model.

**Figure 6:**
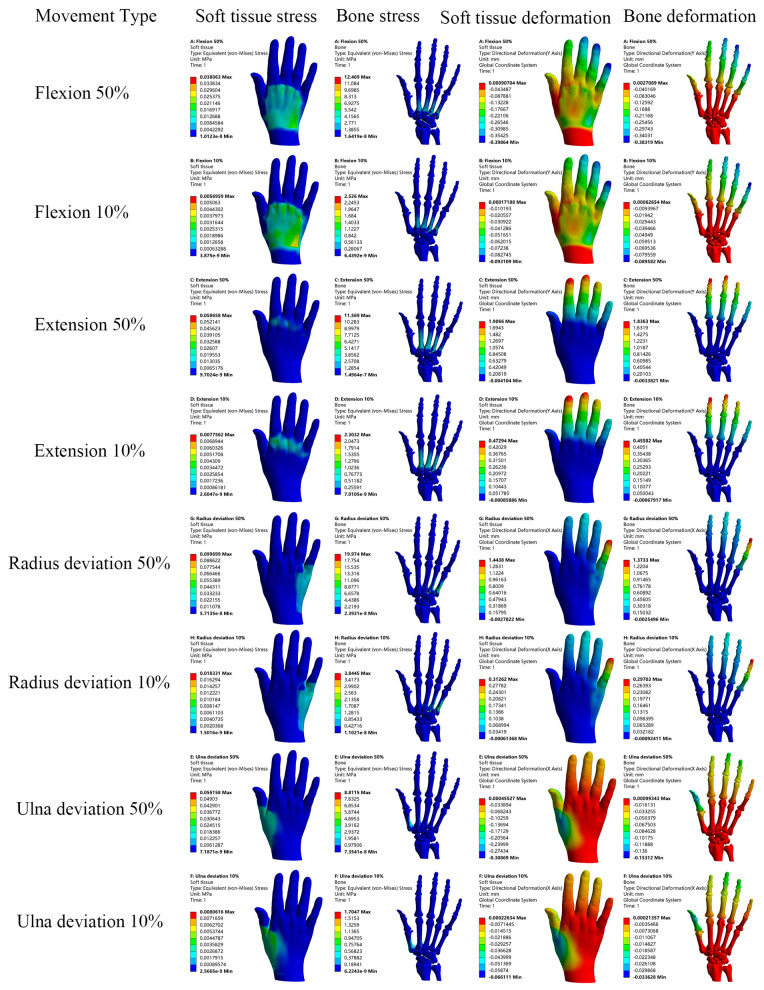
Stress and displacement cloud of RA hand model.

**Figure 7:**
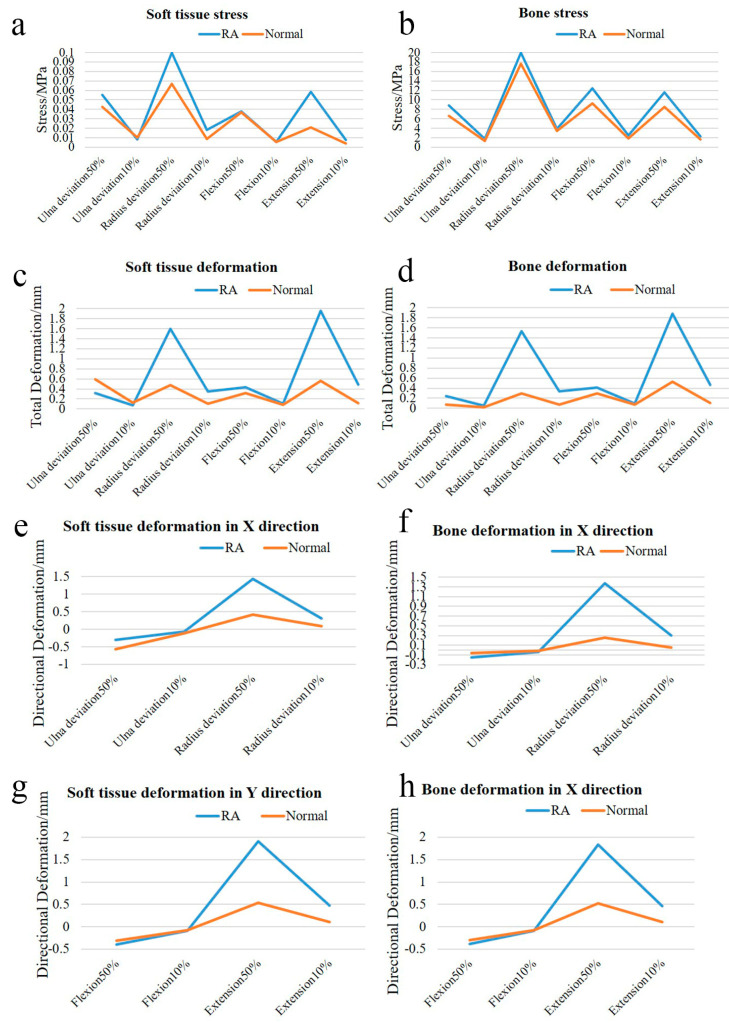
Stress and displacement line graphs for normal and RA models. (**a**,**b**) show the stress line graphs of soft tissues and bones in the four motions of the normal and RA models, (**c**,**d**) show the total displacement line graphs of soft tissues and bones in the four motions of the normal and RA models, (**e**,**f**) show the X-direction displacement line graphs of soft tissues and bones in the ulnar deviation and radial deviation of the normal and RA models, (**g**,**h**) show the Y-direction displacement line graphs of soft tissues and bones in the flexion and extension of the normal and RA models.

Loads were applied to both the normal and RA hand models to simulate four motions: flexion, extension, radial deviation, and ulnar deviation. The resulting stress, total displacement, and directional displacement cloud maps of the soft tissues and bones are presented in Figure 5 and Figure 6. In this analysis, directional displacements in the Y-direction were observed during flexion and extension, while directional displacements in the X-direction were observed during radial and ulnar deviation, in accordance with the direction of load application. In order to compare and analyze the cloud data and distribution differences between the normal and RA hand models more intuitively, the data were tabulated and the related line chart was obtained, as shown in Figure 7.

After conducting a detailed analysis of the data, it was revealed that the RA hand experienced significantly greater stress and displacement compared to the normal hand under identical loading conditions and constraints. As shown in Figure 7a–d, the peak stress in the RA hand was generally 3.22–183.21% higher than that in the normal hand during simulated hand movements. Similarly, Figure 7e–h demonstrated that the peak displacement in the RA hand was consistently 28.81–124.23% higher than that in the normal hand. A literature review confirmed that multiple clinical studies [25,26] and finite element analyses [16,17] have validated the conclusion that the peak stress and displacement of the RA hand are significantly elevated compared to those in the normal hand. These studies employed imaging techniques and biomechanical modeling to diagnose RA-related joint damage, such as bone erosion and joint destruction, followed by disease activity scoring for patients’ hands. The findings indicated that RA scores were substantially higher than those of healthy controls, further supporting the biomechanical differences between RA-affected and normal hands. Additionally, healthy and RA wrist models were developed based on the pathological characteristics of RA, and loads were applied to both. The results showed that the contact stress of the RA model was 3 times higher than that of the normal model, with the RA wrist demonstrating greater extension. These outcomes are consistent with previous literature reviews, suggesting that RA patients experience more pain and discomfort than healthy individuals during daily activities. This phenomenon may be attributed to bone erosion and soft tissue damage in RA patients, leading to structural fragility and susceptibility to stress concentration. The RA finite element model in this study was pretreated to simulate complete cartilage degradation, taking into account the relaxation characteristics of ligaments, which resulted in a significant increase in both peak stress and displacement. Moreover, by analyzing the displacement trend of the normal hand in Figure 7c,d, it is evident that the displacement curve fluctuates less, indicating that the normal hand is more stable due to the combined effects of cartilage and ligaments, which provide a buffering effect against external loads. Subsequently, it is necessary to individually design the orthosis and conduct simulation analysis between the orthosis and RA hand virtual wear. The data obtained in this section can provide a reference for the finite element analysis of the orthosis and verify the effectiveness of the orthosis.

### 3.4. Biomechanical Analysis of Orthotics and the RA Hand

**Figure 8:**
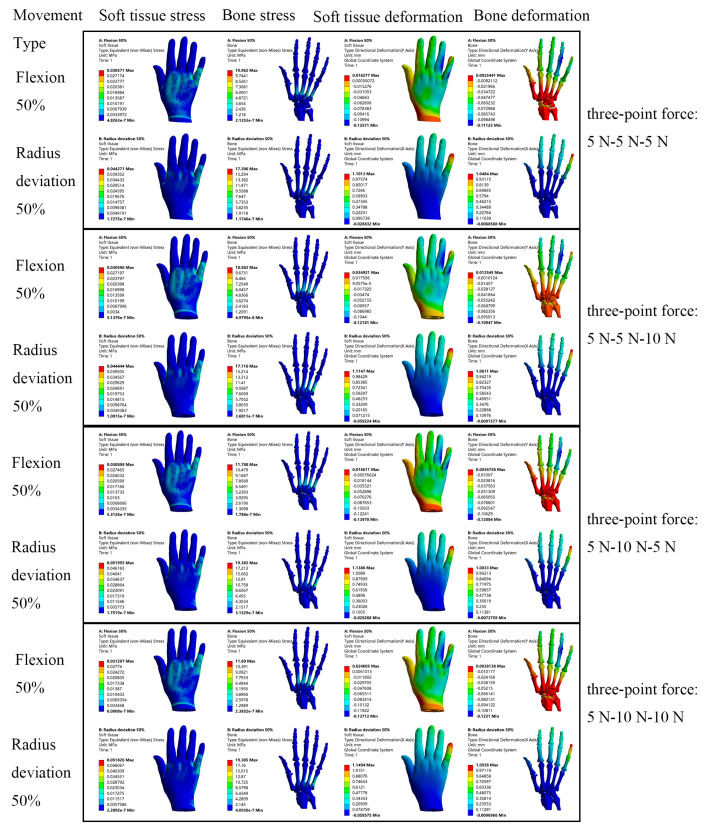
Orthopedic effects of different three-point forces.

**Figure 9:**
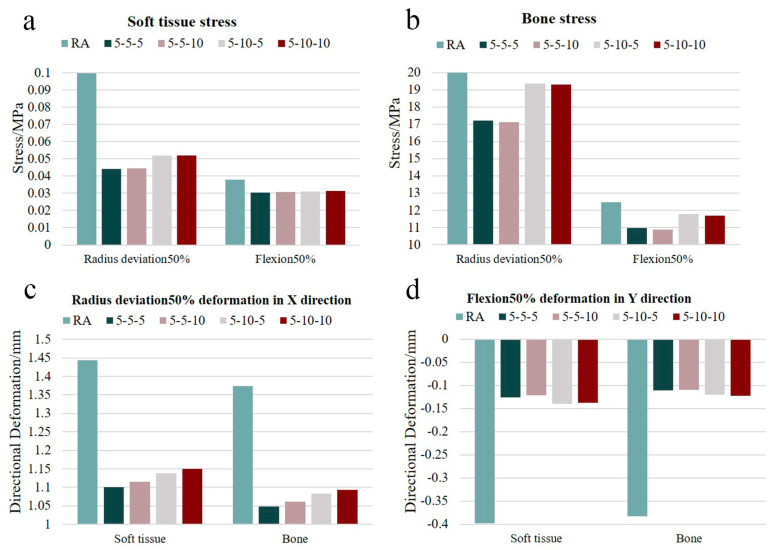
Stress and displacement histogram at different corrective forces after virtual wearing of the orthosis. (**a**,**b**) show the stress histograms of soft tissue and bone under 50% radial deviation and 50% flexion movements for the RA model alone and for the RA model virtually wearing the orthosis and applying four different orthopedic forces, and (**c**,**d**) show the X- or Y-direction displacement histograms of soft tissue and bone under 50% radial deviation and 50% flexion movements for the RA model alone and for the RA model virtually wearing the orthosis and applying four different orthopedic forces.

When the patient wears the hand orthosis, the application of the three-point force is mainly adjusted by the tightness of the Velcro. Since the force exerted between the soft tissues and the orthosis is mutual, the three-point force is applied to the inner surface of the orthosis during its virtual usage. The magnitude of the orthopedic force was set based on the literature and the practical experience of orthotists [23]. In this study, the three-point force was applied at the ulnar side of the fifth proximal interphalangeal joint, the ulnar side of the metacarpophalangeal joint, and the radial side of the radial carpal joint with the following configurations: (1) 5 N-5 N-5 N, (2) 5 N-5 N-10 N, (3) 5 N-10 N-5 N, and (4) 5 N-10 N-10 N. The stress and displacement clouds of soft tissue and bone of the hand under different orthopedic forces can be obtained, as shown in Figure 8. The data obtained from the cloud diagrams were processed and tabulated to generate the corresponding histograms, as shown in Figure 9.

The bar chart in Figure 9 demonstrates the effects of three-point force application (pre- and post-intervention) and the impact of different three-point force magnitudes on stress and displacement in the rheumatoid arthritis hand. The results show that, compared with the direct force on the RA hand model, the peak value of stress and displacement decreased after applying different three-point forces on the virtual orthosis. As shown in Figure 9a,b, during simulated hand movement, the peak stress of the RA hand with the virtual orthosis was generally reduced by 3.05% to 55.60% compared to the condition without the orthosis. Similarly, Figure 9c,d shows that the peak displacement of the RA hand wearing orthosis was generally reduced by 20.35% to 71.43% compared with direct force application. This suggests that when RA patients perform daily hand activities, wearing the orthosis can alleviate hand discomfort and reduce pain. In addition, observing the trend of the bar chart, the different magnitudes of the three-point force applied had varying orthopedic effects. The graph shows that the correction was relatively best when a three-point force of 5 N-5 N-5 N or 5 N-5 N-10 N was applied. Based on these orthopedic outcomes, it is evident that this hand orthosis not only alleviates patient pain but also provides valuable theoretical data for subsequent orthosis research.

## 4. Discussion

The establishment of accurate three-dimensional finite element models is fundamental for studying the anatomy of the wrist joints, investigating the etiology of diseases, selecting treatment methods, and verifying the effectiveness of interventions [27]. Compared to cadaveric weight-bearing studies, finite element analysis presents advantages such as non-invasiveness and enhanced reproducibility. This method allows for the non-invasive reconstruction and mechanical analysis of extremely complex component structures, loads, and material mechanical properties, and it has been increasingly applied to the mechanical study of joints with diverse tissues and intricate structures, such as the wrist joint [28]. Carrigan S. D. et al. developed a wrist model incorporating bone, cartilage, and ligamentous tissues, representing a significant advancement in biomechanical modeling as it is the first to include all eight carpal bones of the wrist along with the associated soft tissues in three dimensions [18]. Gíslason M. K. et al. constructed a finite element model of the wrist, addressing critical issues related to bone geometry, constraints, and contact modeling, and subsequently observed load transfer characteristics by creating a stress map of the wrist bones [29]. Bajuri et al. developed a rheumatoid arthritis model by constructing cartilage based on the anatomy of available joints and simulating ligaments through mechanical connections. A comprehensive literature review indicated that this is the first study to simulate the pathological conditions of rheumatoid arthritis in the wrist joint [17].

The finite element method (FEM) has been widely applied in the study of natural biomaterials, such as bones and soft tissues. In recent years, driven by advancements in computational mechanics and materials science, FEM has also been increasingly utilized for investigating synthetic and composite biomaterials. These studies have significantly enhanced the understanding of the mechanical behavior of biomaterials and provided robust support for the design and application of novel biomaterials. For instance, Fada R et al. combined experimental and FEM simulations to investigate the optimization of mechanical properties in porous bone scaffolds for orthopedic applications. Their work clarified the influence of porosity and strontium nitrate nanoparticles (NPs) on the mechanical performance of calcium phosphate (CaP) bone cement, offering a theoretical foundation for personalized bone scaffold design [30]. Kurtz S M et al. employed FEM to analyze the mechanical response of lumbar total joint replacement (L-TJR) under various misalignment conditions. Their results demonstrated that contact stress, von Mises stress, and effective strain in L-TJR under reasonable misalignment conditions were lower than baseline values in worst-case scenarios. Through FEM validation, they confirmed the mechanical stability of L-TJR under misalignment, providing scientific evidence for clinical safety [31]. Coronary stents, critical medical devices for treating vascular blockages, rely heavily on the mechanical properties of their materials to ensure effective expansion and long-term stability. Volegov P S et al. developed a two-level elastoviscoplastic model to study the internal structure of coronary stent materials and their behavior during deformation. Their research revealed the impact of grain structure size and deformation direction on the mechanical performance of stents, delivering theoretical insights for optimizing stent design and material selection [32].

Therefore, this study utilized 3D modeling software Mimics 19.0, Geomagic Studio 2013, SolidWorks 2021 and finite element analysis software ANSYS Workbench 2021 to develop accurate 3D hand models that incorporate bones, cartilage, soft tissues, ligaments, and other associated anatomical structures. The hand model is consistent with the anatomical configuration of the human hand. Then, the biomechanical behavior of rheumatoid arthritis hand was studied by finite element analysis using a healthy hand model as control. The same loads were applied to both the healthy and RA hand models to simulate four hand motions: flexion, extension, radial deviation, and ulnar deviation. This approach enabled the assessment of maximum stresses, maximum displacements, and corresponding maximum directional displacements of the soft tissues and bones. A detailed analysis of the results revealed that, under identical loading conditions and constraints, the RA hand exhibited significantly higher stress and displacement compared to the healthy hand. As illustrated in Figure 7, during simulated hand motions, the peak stress in RA hands was elevated by 3.22% to 183.21% relative to normal hands. Similarly, the peak displacement in RA hands surpassed that of normal hands by 28.81% to 124.23%. This discrepancy may be attributed to the damage to articular cartilage in the hands of RA patients, which impairs proper load transmission and increases the likelihood of stress concentrations. Moreover, due to ligament laxity in RA patients, they are more susceptible to hyperflexion, hyperextension, and ulnar deviation when performing the same hand movements.

At present, the effectiveness of hand orthotics is mostly verified by clinical function evaluation, such as clinical efficacy score, and the data obtained are subjective. Tijhuis et al. conducted a comparative study on the short-term utility and clinical outcomes of two types of wrist orthoses: off-the-shelf and custom-made. Ten RA patients were randomly assigned to use each type of orthosis for a two-week period and completed a questionnaire to assess their experience. The study found no significant difference in effectiveness between the two orthotic types [33]. However, this conclusion may be influenced by the brief duration of orthotic use and the inherent subjectivity of the questionnaire. Additionally, the participants’ abilities to accurately describe their experience may have been limited. Sadura-Sieklucka T. et al. conducted a study involving 104 women with rheumatoid arthritis (RA) who wore a wrist stabilizer, with a control group consisting of 40 healthy women of the same age. The results demonstrated that the use of wrist stabilizers in patients with RA is beneficial, leading to improvements in hand strength and dexterity while reducing pain [7]. Aranceta-Garza A. et al. investigated, tested, and compared the effectiveness and functionality of 10 commercially available wrist and hand orthoses with respect to wrist mobility and grip strength. The study revealed that, based on the 2400 tests conducted, the wrist-hand orthoses were inadequate for successfully managing situations requiring pain-related movement restrictions [34]. The reasons for this inadequacy were analyzed and may be attributed to design factors of the orthoses, such as geometry and construction materials. These findings underscore the necessity for redesigning hand orthoses to better meet user needs.

In order to more objectively assess the efficacy of hand orthoses, this study developed personalized hand orthoses for RA patients based on their clinical conditions, hand anatomical features, and the principle of three-point force rehabilitation and correction. After that, the wearing of virtual orthopedic devices for hand movement was simulated on patients. At the same time, three-point forces of different sizes were applied to the ulnar of the fifth proximal interphalangeal joint, the ulnar of the metacarpal and phalangeal joints, and the radial carpal joints, respectively, to observe the orthopedic effect of RA hand. The results showed that, compared to direct force application on the RA hand model, the virtual application of an orthosis significantly reduced peak stress and displacement in the RA hand. As illustrated in Figure 9, during simulated hand motion, the peak stress in the RA hand with the orthosis was reduced by 3.05–55.60% compared to the condition without the orthosis. Similarly, the peak displacement in the orthosis-equipped RA hand decreased by 20.35–71.43% relative to direct force loading. In daily activities, rheumatoid arthritis patients experience less hand pain when wearing orthoses. This effect may be attributed to the combined mechanisms of the orthosis and three-point forces, which enhance stress distribution, optimize load transfer pathways, and, thereby, mitigate tissue damage while promoting recovery. This study emphasizes personalized orthosis design tailored to individual RA patients’ disease severity and hand morphology, ensuring optimal fit. The orthosis provides additional support, effectively disperses mechanical stress, and redirects load transfer pathways during hand movements, reducing localized stress concentrations and pain. Furthermore, three-point forces facilitate soft tissue remodeling through sustained low-magnitude mechanical loading, alleviate high-pressure zones in deformed joints, and reduce the risk of secondary injuries in adjacent joints caused by abnormal loads. Different magnitudes of applied three-point forces led to varying orthopedic effects. Among the four applied orthopedic forces of (1) 5 N-5 N-5 N, (2) 5 N-5 N-10 N, (3) 5 N-10 N-5 N, and (4) 5 N-10 N-10 N, the configurations of 5 N-5 N-5 N and 5 N-5 N-10 N produced relatively optimal orthopedic effects.

Based on the aforementioned research findings, personalized configurations of hand orthoses have been shown to effectively alleviate pain and correct deformities in patients with rheumatoid arthritis. Furthermore, these configurations can provide valuable theoretical data for subsequent treatment strategies for RA. Biomechanical analysis via finite element methods facilitates the virtual application of orthoses to the RA patient hand model, making the effects more intuitive and controllable, thereby enhancing the research process.

Although this study validates the rationality of hand orthosis design and the efficacy of corrective treatment from a biomechanical perspective through finite element analysis, several aspects still require further improvement. Specifically, the clinical feasibility should be enhanced, and more comprehensive patient case studies should be incorporated. Therefore, in future studies, in-depth collaboration with hospitals will be conducted to detect bone density via dual-energy X-ray absorptiometry (DXA) and quantitative CT (QCT), while measuring cartilage destruction using magnetic resonance imaging (MRI) and ultrasound and evaluating ligament relaxation through physical examinations and ultrasound elastography. Based on clinically measured patient data, model parameters such as elastic modulus and Poisson’s ratio will be modified. The model simulation will then be associated with orthotic intervention to verify the orthotic effect on patients. Different case studies can be created by adjusting the model parameters according to individual patient characteristics, such as age, gender, bone structure, and disease severity. Optimization of orthotic design will also be pursued. Orthotics tailored to the patient’s condition and hand characteristics have proven effective. Stress and strain data from simulation results can guide further optimization of orthotic design to enhance treatment outcomes. For instance, high-stress areas can be locally reinforced, and the shape and wearing mode of orthotics can be optimized. Additionally, the selection of orthopedic materials should be rationalized. Materials for rheumatoid arthritis orthotics need to be flexible, viscoelastic and biocompatible. As a rigid thermoplastic material, polylactic acid (PLA) may cause discomfort and even increase joint pressure in patients, resulting in deviations between simulation results and clinical orthopedic effects, thus weakening the guiding value of the research for clinical practice. Materials such as thermoplastic polyurethane (TPU) and silicone resin offer good flexibility and durability, which better meet the requirements of orthotics in clinical use, thereby improving the practicability and credibility of the research. Future studies can alter the stiffness value of the orthotic model and combine finite element analysis with 3D printing technology to compare and analyze simulation results with actual clinical applications, thus designing more competitive orthotics.

## 5. Conclusions

The hand is a highly complex and flexible structural mechanism. A biomechanical analysis of the hands of both healthy individuals and patients with rheumatoid arthritis has demonstrated that, in contrast to normal hands, those affected by RA exhibit higher stress and displacement values. Subsequently, based on the patient’s condition, the three-point mechanical correction principle was employed to design an orthosis tailored to fit the patient’s hand. After virtual application, the maximum values of stress and displacement in the hands of RA patients were found to be reduced. Additionally, the orthopedic outcomes varied depending on the magnitude of the applied three-point orthopedic force. These results indicate that the orthosis can alleviate pain in patients and provide valuable theoretical data for subsequent orthosis research. By employing finite element modeling methods for analysis, a deeper understanding of the biomechanical outcomes of the hand can be achieved. Furthermore, based on the objectives and findings of this study, the orthosis can be promptly adjusted and refined.

## Figures and Tables

**Table 1:** Material properties.

Model	Young’s Modulus (MPa)	Poisson’s Ratio
Cortical bone	18,000.00	0.20
Cancellous bone	100.00	0.25
RA cortical bone	12,000.00	0.20
RA cancellous bone	33.00	0.25
Cartilage	10.00	0.45
Soft tissue	1.15	0.49
Orthotics	1300.00	0.46

**Table 2:** Loads applied by the four motions.

Movement Type	Applied 100% Load (N)	Applied 50% Load (N)	Applied 10% Load (N)
Flexion	147.4	73.7	14.7
Extension	82.8	41.4	8.3
Radius deviation	112.8	56.4	11.3
Ulna deviation	97.8	48.9	9.8

**Table 3:** Model validation results.

Data Source	Radius	Ulna
Literature (Normal) [22]	78.7–92.8%	7.2–21.3%
Normal	81.5%	18.5%
RA	76.1%	23.9%

## Data Availability

The original data are available from the corresponding author upon an appropriate request.
